# Association between family history, early growth and the risk of beta cell autoimmunity in children at risk for type 1 diabetes

**DOI:** 10.1007/s00125-020-05287-1

**Published:** 2020-10-07

**Authors:** Danièle Pacaud, Anita M. Nucci, David Cuthbertson, Dorothy J. Becker, Suvi M. Virtanen, Johnny Ludvigsson, Jorma Ilonen, Mikael Knip, the TRIGR investigators

**Affiliations:** 1 Department of Pediatrics, Alberta Children’s Hospital, University of Calgary, Calgary, AB Canada; 2 Department of Nutrition, Georgia State University, Atlanta, GA USA; 3 Pediatrics Epidemiology Center, University of South Florida, Tampa, FL USA; 4 Division of Endocrinology, University of Pittsburgh and UPMC Children’s Hospital of Pittsburgh, Pittsburgh, PA USA; 5 Public Health Promotion Unit, National Institute for Health and Welfare, Helsinki, Finland; 6 Faculty of Social Sciences/Health, Tampere University, Tampere, Finland; 7 Center for Child Health Research, Tampere University, Tampere, Finland; 8 Research, Development and Innovation Centre, Tampere University Hospital, Tampere, Finland; 9 Crown Princess Victoria Children’s Hospital, Region Östergötland and Division of Pediatrics, Department of Clinical Experimental Medicine, Linkoping University, Linkoping, Sweden; 10 Immunogenetics Laboratory, Institute of Biomedicine, University of Turku, Turku, Finland; 11 Clinical Microbiology, Turku University Hospital, Turku, Finland; 12 Pediatric Research Center, Children’s Hospital, University of Helsinki and Helsinki University Hospital, Helsinki, Finland; 13 Research Program for Clinical and Molecular Metabolism, Faculty of Medicine, University of Helsinki, Helsinki, Finland

**Keywords:** Beta cell autoimmunity, Birthweight, Familial risk, Father, Genetic risk, Linear growth, Mother, Proband, Type 1 diabetes

## Abstract

**Aims/hypothesis:**

The aim of this work was to examine the relationship between family history of type 1 diabetes, birthweight, growth during the first 2 years and development of multiple beta cell autoantibodies in children with a first-degree relative with type 1 diabetes and HLA-conferred disease susceptibility.

**Methods:**

In a secondary analysis of the Trial to Reduce IDDM in the Genetically at Risk (TRIGR), clinical characteristics and development of beta cell autoantibodies were compared in relation to family history of type 1 diabetes (mother vs father vs sibling) in 2074 children from families with a single affected family member.

**Results:**

Multiple autoantibodies (≥2 of 5 measured) developed in 277 (13%) children: 107 (10%), 114 (16%) and 56 (18%) born with a mother, father or sibling with type 1 diabetes, respectively (*p* < 0.001). The HR for time to multiple autoimmunity was 0.54 (95% CI 0.39, 0.75) in offspring of affected mothers (*n* = 107/1046, *p* < 0.001) and 0.81 (95% CI 0.59, 1.11) (*n* = 114/722, *p* = 0.19) in offspring of affected fathers, compared with participants with a sibling with type 1 diabetes (comparator group *n* = 56/306). The time to the first autoantibody present (to insulin, GAD, tyrosine phosphatase-related insulinoma-associated 2 molecules, islet cell or zinc transporter 8) was similar in the three groups. Height velocity (*z* score/year) in the first 24 months was independently associated with developing multiple antibodies in the total cohort (HR 1.31 [95% CI 1.01, 1.70], *p* = 0.04). A higher birthweight in children born to an affected mother vs affected father or an affected sibling was not related to the risk of multiple autoimmunity.

**Conclusions/interpretation:**

The risk of developing multiple autoantibodies was lower in children with maternal type 1 diabetes. For the whole group, this risk of developing multiple autoantibodies was independent of birthweight but was greater in those with increased height velocity during the first 2 years of life. However, the risk associated with paternal type 1 diabetes was not linked to differences in birthweight or early growth.

**Trial registration:**

ClinicalTrials.gov NCT00179777

Graphical abstract
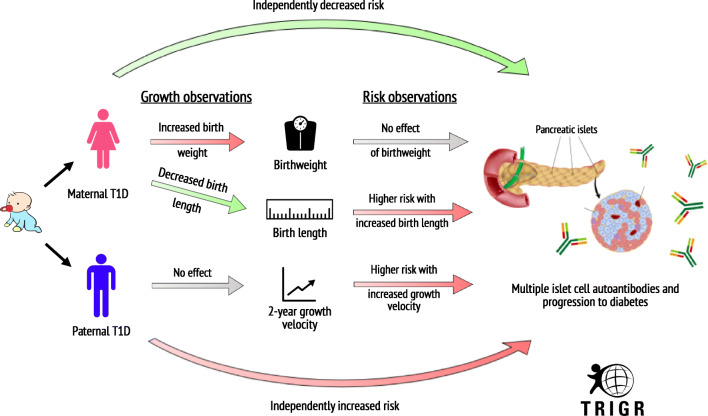

**Electronic supplementary material:**

The online version of this article (10.1007/s00125-020-05287-1) contains peer-reviewed but unedited supplementary material, which is available to authorised users.

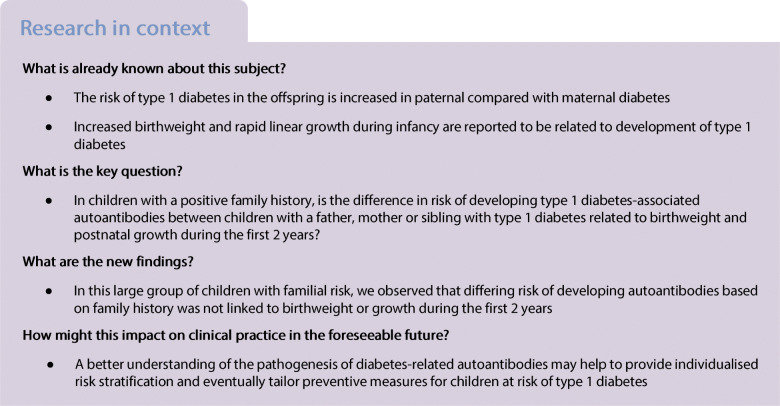



## Introduction

Type 1 diabetes is one of the most prevalent chronic diseases in childhood. Its incidence is rapidly increasing worldwide [1–5]. Type 1 diabetes is caused by a progressive but heterogeneous autoimmune destruction of the beta cells resulting in lack of insulin. Both genetic and environmental factors contribute to this process, although its pathophysiology is poorly understood [6]. Historically, the risk of diabetes in the offspring of fathers with type 1 diabetes has been reported as being higher than that in the offspring of mothers with type 1 diabetes or in those with siblings who have type 1 diabetes [7]. Many researchers have examined the link between birthweight and risk of either type 1 diabetes or beta cell autoimmunity with variable results. This may reflect the heterogeneity of the sample size, study population and methodology used. However, a large meta-analysis of 29 studies predominantly from European centres, found that the risk of type 1 diabetes was significantly higher in children with greater birthweights [8]. Larger studies, looking specifically at individuals genetically at risk for type 1 diabetes, observed that greater birthweight is related to development of type 1 diabetes [9, 10] or beta cell autoimmunity [10] and that this pattern is similar across different geographical regions. Furthermore, some studies that were able to prospectively observe children with genetic risk for type 1 diabetes have identified rapid linear growth during infancy as a risk factor for the development of beta cell autoimmunity and type 1 diabetes [11–14].

Although The Environmental Determinants of Diabetes in the Young (TEDDY) study group recently reported that family history is not a significant risk factor for the progression to diabetes in individuals who develop multiple autoantibodies [15], their previous reports focusing on growth and risk beta cell autoimmunity, did not account for first-degree relative status in their analysis [16]. However, a recent analysis examining the effect of interactions between genetic and environmental factors on the development of islet cell autoimmunity and diabetes indicated that family history (father or sibling vs no family history) significantly influenced both, while weight at 12 months only affected development of autoimmunity but not type 1 diabetes [17]. To our knowledge, none of the studies looking at birthweight, infant growth, HLA typing and risk of beta cell autoimmunity evaluated possible differences in risk linked to birthweight, infant growth and autoimmunity associated with the proband (father, mother or sibling). In fact, many of these studies excluded maternal diabetes from the analysis in order to avoid the confounding factor associated with maternal metabolic control and birthweight. In this study we examined the relationship between the family history of type 1 diabetes (mother vs father vs sibling), birthweight and initial 2 year growth and autoimmunity in a cohort of 2074 participants with one first-degree relative with type 1 diabetes in the Trial to Reduce IDDM in the Genetically at Risk (TRIGR) study (ClinicalTrials.gov registration no. NCT00179777).

## Methods

### Study design and participants

The TRIGR study was an international double-blinded clinical trial designed to determine whether weaning to a hydrolysed infant formula vs a conventional cow’s milk-based formula reduces the cumulative incidence of diabetes-associated autoantibodies and the incidence of diabetes in young children with a first-degree relative with type 1 diabetes and increased HLA-conferred genetic risk. The trial was approved in all participating TRIGR centres by the Ethics Institutional Review Boards or Committees of Human Experimentation and informed consent was obtained for each participating child. A full description of the TRIGR study design has been published previously [18]. Children at increased risk for developing type 1 diabetes were recruited for the trial before or at birth between May 2002 and January 2007 (*n* = 2159) and were monitored until February 2017, when all children were at least 10 years of age, for the frequency of beta cell autoantibodies and/or the development of type 1 diabetes. For the purpose of this secondary analysis, we excluded 73 children with more than one first-degree family member with type 1 diabetes: ten with mother and sibling; 33 with father and sibling; 29 with both mother and father; and one with a mother, father and sibling with type 1 diabetes. Another four were excluded because of missing family relationship status and eight were excluded due to missing antibody data.

#### Data collection

Only data collected during the conduct of the original TRIGR study was used for this secondary analysis. Demographic characteristics, including sex, race, ethnicity and data on type 1 diabetes in first-degree relatives, were obtained at the time of enrolment or birth. HLA genotyping to determine eligibility was performed [19]. Anthropometric indices were obtained at birth and at regular intervals (values at 3, 6, 9, 12, 18 and 24 months of age are included in this analysis). Weight velocity (kg/year) and length/height velocity (cm/year), as well as corresponding *z* score values calculated from 2000 CDC growth charts [20], were recorded for the first 2 years of life. BMI (kg/m^2^) was calculated at 2 years of age. The definition of exclusive breastfeeding was intake of only breast milk (banked or mother’s own), supplementary vitamins or minerals or water. The presence of beta cell autoantibodies was determined in a central laboratory (Scientific Laboratory, Children’s Hospital, University of Helsinki, Helsinki, Finland) at birth and at 3, 6, 9, 12, 18 and 24 months of age and annually thereafter. Islet cell autoantibodies (ICA) were measured using indirect immunofluorescence with a cut-off of 2.5 JDRF units [19] and GAD autoantibodies (GADA), tyrosine phosphatase-related insulinoma-associated 2 molecule autoantibodies (IA-2A), insulin autoantibodies (IAA) and zinc transporter 8 autoantibodies (ZnT8A) were analysed with specific radiobinding assays with cut-off limits of 5.36 RU, 0.77 RU, 2.80 RU and 0.61 RU, respectively [21]. Development of an autoantibody was defined as the first occurrence of a result above the specified cut-off limits. The disease-specific sensitivity and specificity for each were previously reported [19, 22]. Maternal antibodies that were placentally transferred, as verified by their decreasing levels and disappearance from the child’s circulation by age 18 months, were not included in the statistical analysis.

### Statistical analysis

We compared demographic, clinical and anthropometric characteristics by family history of type 1 diabetes (mother vs father vs sibling) univariately using the χ^2^ test for categorical variables and ANOVA for continuous variables. Cox proportional hazard univariate model was used to analyse the relationship between family history of type 1 diabetes (mother vs father vs sibling) and time to multiple autoimmunity (defined as two or more autoantibodies), positivity for GADA, IAA, IA-2A, ICA and ZnT8A and development of clinical type 1 diabetes. Multivariate Cox proportional hazard regression models were created to predict time to antibodies (multiple autoantibodies, GADA, IAA, IA-2A, ICA or ZnT8A) and the development of clinical type 1 diabetes in each family history group (mother vs father vs sibling). The following variables thought to be clinically important were selected a priori: country of birth, sex, HLA risk group, mode of delivery, birth length, birthweight, treatment group, height velocity in the first 24 months of life (cm/year), weight velocity in first 24 months of life (*z* score), BMI *z* score at 24 months of life, infant diet in the first days of life, and length of exclusive breastfeeding. These were then included in a stepwise model when found to be statistically significant. Using the same covariates listed above, general linear regression models (GLMs) were used to examine the difference between the three groups of origin of the proband in linear growth both in cm/year and *z* score/year. All HRs are presented with the sibling group as the reference group. A *p* value of <0.05 was considered statistically significant for the comparisons between the three groups. All participants with missing data specified as part of a particular analysis were omitted from that analysis. No imputation of data was performed. All analyses were conducted using SAS version 9.4, USA. All results presented in the Results section are new and were not part of previous TRIGR reports.

## Results

### Beta cell autoimmunity and relationship to family history of type 1 diabetes

**Fig. 1:**
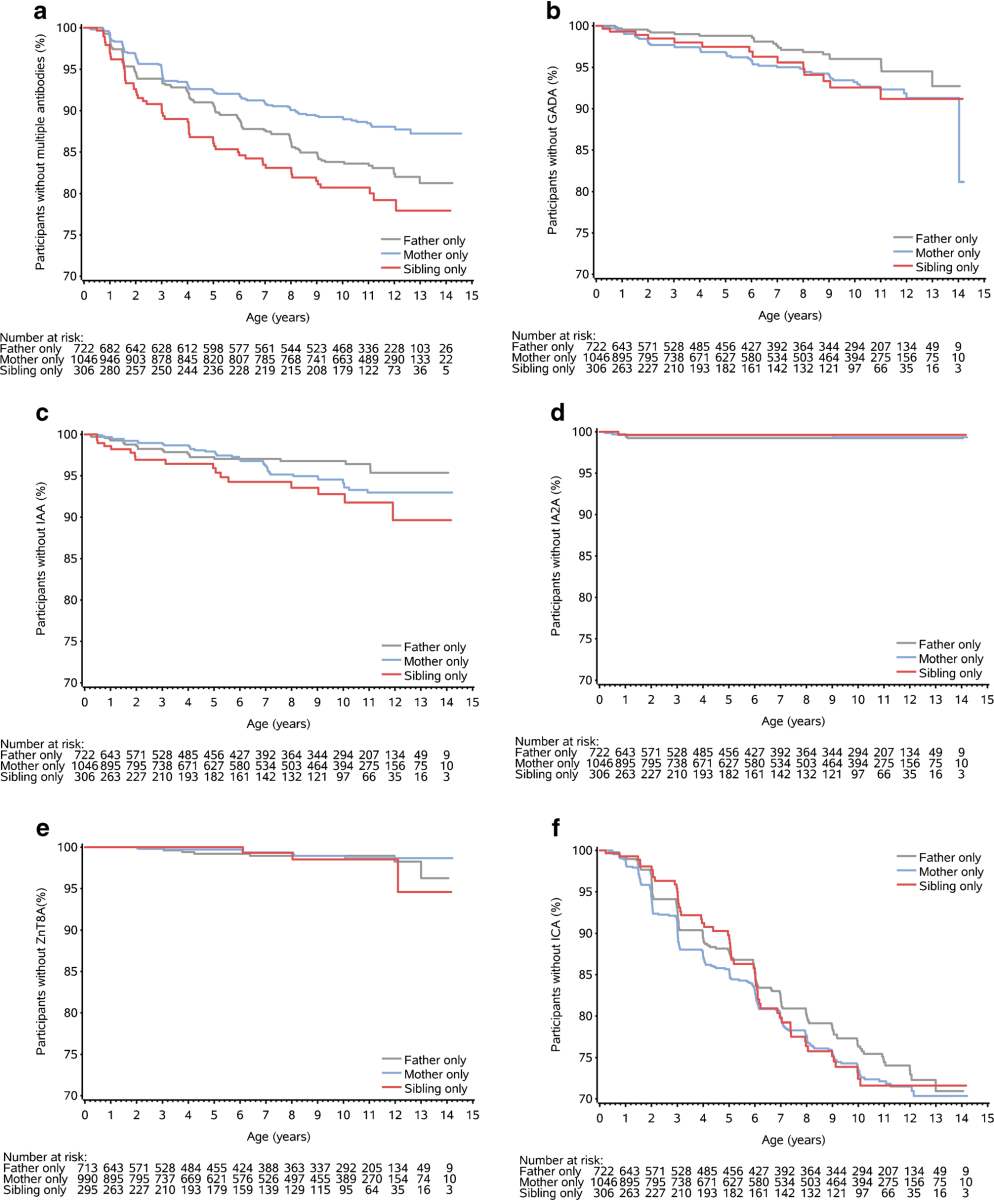
Time to presence of autoantibodies by family history (mother vs father vs sibling): multiple autoantibodies defined as two or more antibodies (logrank χ^2^ 16.2, *p* < 0.001) (**a**); GADA as first antibody (logrank χ^2^ 4.8, *p* = 0.090) (**b**); IAA as first antibody (logrank χ^2^ 5.5, *p* = 0.064) (**c**); IA-2A as first antibody (logrank χ^2^ 0.55, *p* = 0.761) (**d**); ZnT8A as first antibody (logrank χ^2^ 0.6, *p* = 0.73) (**e**); and ICA as first antibody (logrank χ^2^ 1.2, *p* = 0.55) (**f**)

The clinical characteristics of the 2074 children with a single first-degree relative with type 1 diabetes are presented in Table 1. During the study period, positive seroconversion occurred in 933 (45%) children, which included 346 (17%) with GADA, 319 (15%) with IAA, 196 (9%) with IA-2A, 724 (35%) with ICA and 171 (8%) with ZnT8A. Multiple autoantibodies (two or more of GADA, IA-2A, IAA, ZnT8A and ICA) developed in 277 (13%) children: 107 (10%), 114 (16%) and 56 (18%) of children born with a mother, father or sibling with type 1 diabetes, respectively. Time to the development of multiple autoantibodies in the child differed significantly between those with an affected mother, father or sibling (time to 10% cumulative incidence [95% CI]: mothers 9.3 years [7.7, 11.3]; fathers 6.3 years [5.2, 7.6]; sibling 5.0 years [3.9, 6.5]) (logrank χ^2^ 16.2, *p <* 0.001) (Fig. 1a). Pairwise comparisons between the three groups indicated that while there was not a significant difference in the progression to multiple autoantibodies between those with an affected father and sibling (logrank χ^2^ 1.7, *p* = 0.189), there was a significant difference between those with an affected mother and, respectively, those with an affected father (logrank χ^2^ 8.7, *p* = 0.003) and those with an affected sibling (logrank χ^2^ 14.0, *p* < 0.001).

**Table 1:** Demographic, clinical and anthropometric characteristics by family history of type 1 diabetes (mother vs father vs sibling)

Variable	Mother (*n* = 1046)	Father (*n* = 722)	Sibling (*n* = 306)	*p* value
Male sex, *n* (%)	547 (52)	391 (54)	156 (51)	NS
Birth order^a^	1 (1)	1 (1)	2 (1)	<0.001
Birthweight, kg	3.65 ± 0.60	3.56 ± 0.49	3.57 ± 0.49	0.004
Birth length, cm	50.9 ± 3.0	51.0 ± 2.4	51.6 ± 2.7	<0.001
Weight at 24 months, cm	12.8 ± 1.6	12.9 ± 1.4	12.9 ± 1.7	0.627
Weight at 24 months, *z* score	0.2 ± 1.1	0.3 ± 1.0	0.2 ± 1.2	0.346
BMI at 24 months, *z* score	0.08 ± 1.15	−0.03 ± 1.04	−0.15 ± 1.17	0.054
Height velocity at 24 months, cm/year	18.3 ± 2.0	18.5 ± 1.9	18.2 ± 1.8	0.053
Height velocity at 24 months, *z* score	−0.04 ± 0.66	0.00 ± 0.61	−0.011 ± 0.56	0.041
^b^HLA risk type, *n* (%)				0.017
HLA risk 1	229 (22)	171 (24)	95 (31)	
HLA risk 2	456 (44)	336 (47)	117 (38)	
HLA risk 3	350 (34)	210 (29)	92 (30)	
HLA risk 4	11 (1)	5 (1)	2 (1)	
Length of exclusive breastfeeding, months	0.7 ± 1.6	1.8 ± 2.2	2.3 ± 2.3	<0.001
Caesarean section, *n* (%)	642 (61.4)	183 (25.3)	74 (24.2)	<0.001
Gestational age, weeks	37.8 ± 1.3	39.8 ± 1.4	39.4 ± 1.4	<0.001
Autoantibodies, *n* (%)				
Multiple	107 (10)	114 (16)	56 (18)	<0.001
IAA	139 (13)	110 (15)	70 (23)	<0.001
GADA	154 (15)	126 (18)	66 (22)	0.015
IA-2A	72 (7)	85 (12)	39 (13)	<0.001
ZnT8A	65 (6)	75 (10)	31 (10)	0.003
ICA	337 (32)	270 (37)	117 (38)	0.034
Type 1 diabetes, *n* (%)	59 (6)	60 (8)	38 (12)	<0.001
Follow-up time, years	9.1 ± 4.3	9.4 ± 4.0	8.7 ± 4.3	0.032

Values are expressed as mean±SD or *n* (%)

^a^Values are expressed as median (IQR)

^b^HLA risk 1, HLA-DQB1*02/DQB1*03:02; HLA risk 2, HLA-DQB1*03:02/x (x not DQB1*02, DQB1*03:01 or DQB1*06:02); HLA risk 3, HLA-DQA1*05-DQB1*02/y (y not DQA1*02:01-DQB1*02, DQB1*03:01, DQB1*06:02 or DQB1*06:03); HLA risk 4, HLA-DQA1*03-DQB1*02/y (y not DQA1*02:01-DQB1*02, DQB1*03:01, DQB1*06:02 or DQB1*06:03)

### First positive beta cell autoantibody and relationship of family member affected by type 1 diabetes

In children developing multiple antibodies, the first was GADA in 52 (19%), IAA in 48 (18%), ICA in 46 (17%), IA-2A in three (1%) and ZnT8A in three (1%). Multiple antibodies occurred in the first positive sample in 119 (44%). Among the 119 children who developed multiple antibodies in the first sample, 59% tested positive for GADA, 52% for IAA, 62% for ICA, 27% for IA-2A and 21% for ZnT8A, respectively. There was no difference in which antibody appeared first between the three groups (*p* = 0.39). Times to each antibody as the first antibody present were similar in all three groups (logrank χ^2^ 0.5–5.5; all *p* > 0.05) (Fig. 1b–f).

### Association between birth and growth variables and affected family member relationship

Despite early differences in mode of delivery, gestational age, birthweight, birth length and duration of exclusive breastfeeding (see Table 1), univariate analysis demonstrated at age 24 months only mild non-significant differences in BMI *z* score (ESM Fig. 1, *p =* 0.054), height velocity (ESM Fig. 2a, *p* = 0.053) between the three groups. However, there was a significant difference between the three groups in height velocity *z* score (ESM Fig. 2b, *p =* 0.041), explained by a wider variation in growth seen in children born to a father with diabetes. GLMs predicting height velocity either as cm/year (Model 1, ESM Table 1) or *z* score per year (Model 2, ESM Table 1) until age 24 months by relative with type 1 diabetes resulted in significant differences: height velocity in cm/year *R*^2^ was 0.246 with *F* (13, 1754) = 44.0; *p* < 0.001, while for height velocity in *z* score/year *R*^2^ was 0.399 with *F* (13, 1754) = 89.5; *p* < 0.001. Variables found to contribute to each model were similar between the two GLM models (birth length, birthweight, mother with type 1 diabetes, region of origin, duration of exclusive breast feeding, being fed only infant formula in the first week of life vs other types of first diet, birth order and gestational age) (ESM Table 1). In both models, the relative with type 1 diabetes showed a statistically significant but minor association: infants of an affected mother explained <1% of the variation in growth. Re-enforcing this point, removing family history as a variable in these models produced remarkably similar results, with *R*^2^ values of 0.237 and 0.391 for the models for height velocity in cm/year and *z* score/year, respectively).

### Association between the development of multiple autoantibodies, family member with type 1 diabetes and first 2 years of physical growth

When Cox proportional hazard stepwise multivariate modelling for time to multiple autoimmunity with an a priori inclusion of family member with type 1 diabetes was used, being born to a mother with type 1 diabetes remained the lowest risk of developing multiple antibodies (HR 0.70 [95% CI 0.49, 1.01], *p =* 0.055) compared with an affected father or sibling (Table 2). The other significant variables retained in this model were HLA risk 1 (HR 1.74 [95% CI 1.33, 2.28], *p <* 0.001), birth length (HR 1.09 [95% CI 1.03, 1.16], *p =* 0.004) and height velocity *z* scores in the first 24 months (HR 1.31 [95% CI 1.01, 1.70], *p =* 0.041).

**Table 2:** HR (95% CI) from univariate and multivariate Cox time to development of multiple autoantibodies model

Variable	Univariate models		Multivariate model	
	HR (95% CI)	*p* value	HR (95% CI)	*p* value
Family history from mother	0.54 (0.39, 0.75)	<0.001	0.70 (0.49, 1.01)	0.055
Family history from father	0.81 (0.59, 1.11)	0.191	0.93 (0.65, 1.33)	0.682
HLA risk 1^a^	1.92 (1.50, 2.45)	<0.001	1.74 (1.33, 2.28)	<0.001
Birth length	1.06 (1.01, 1.11)	0.012	1.09 (1.03, 1.16)	0.004
Height velocity (*z* score)	1.03 (0.84, 1.27)	0.758	1.31 (1.01, 1.70)	0.041
Weight velocity (*z* score)	1.02 (0.84, 1.23)	0.880		
Male sex	1.27 (1.00, 1.61)	0.054		
Birthweight	1.08 (0.87, 1.33)	0.494		
Gestational age, weeks	1.05 (0.98, 1.13)	0.168		
Region of origin				
Northern Europe	1.11 (0.85, 1.45)	0.449		
Central Europe	1.10 (0.76, 1.58)	0.614		
Vaginal delivery	1.24 (0.97, 1.58)	0.084		
Treatment group assignment casein hydrosylate formula	1.13 (0.89, 1.43)	0.314		
Exclusive breastfeeding duration	1.05 (1.00, 1.11)	0.071		
Birth order	1.06 (0.95, 1.18)	0.314		

^a^HLA-DQB1*02/DQB1*03:02

### Association between risk of type 1 diabetes and relationship of affected family member

The time to the development of type 1 diabetes was compared between the three groups of family member with type 1 diabetes. Univariate analyses indicated that there was an overall difference between the groups (time to 5% cumulative incidence [95% CI]: mothers 10.0 years [7.7, 12.9]; fathers 6.9 years [5.4, 8.9]; sibling 4.3 years [3.1, 5.9]) (logrank χ^2^ 17.2, *p* < 0.001) (ESM Fig. 3). When Cox proportional hazard stepwise multivariate modelling for time to development of type 1 diabetes with an a priori inclusion of family member with type 1 diabetes was used, those with a mother, compared with an affected father or sibling, with type 1 diabetes remained at lowest risk of developing type 1 diabetes (HR 0.58 [95% CI 0.36, 0.93], *p =* 0.02). The other significant variables retained in this model were HLA risk 1 (HR 2.21 [95% CI 1.54, 3.16)], *p <* 0.001) and height velocity *z* score in the first 24 months (HR 1.41 [95% CI 1.10, 1.82], *p <* 0.01) (Table 3).

**Table 3:** HR (95% CI) from univariate and multivariate Cox time to the development type 1 diabetes model

Variable	Univariate models		Multivariate model	
	HR (95% CI)	*p* value	HR (95% CI)	*p* value
Family history from mother	0.43 (0.29, 0.65)	<0.001	0.58 (0.36, 0.93)	0.023
Family history from father	0.62 (0.41, 0.93)	0.021	0.67 (0.41, 1.09)	0.104
HLA risk 1^a^	2.35 (1.71, 3.24)	<0.001	2.21 (1.54, 3.16)	<0.001
Height velocity (*z* score)	1.38 (1.07, 1.78)	0.012	1.41 (1.10, 1.82)	0.007
Birth length	1.01 (0.95, 1.07)	0.732		
Weight velocity, *z* score	1.40 (1.06, 1.84)	0.017		
Male sex	1.07 (0.78, 1.46)	0.682		
Birthweight	0.99 (0.74, 1.31)	0.930		
Gestational age, weeks	1.03 (0.94, 1.14)	0.539		
Region of origin				
Northern Europe	1.09 (0.77, 1.56)	0.625		
Central Europe	1.02 (0.62, 1.67)	0.936		
Vaginal delivery	1.28 (0.93, 1.77)	0.132		
Treatment group assignment, casein hydrosylate formula	1.06 (0.78, 1.45)	0.717		
Exclusive breastfeeding duration	1.05 (0.98, 1.13)	0.183		
Birth order	1.08 (0.93, 1.25)	0.307		

^a^HLA-DQB1*02/DQB1*03:02

## Discussion

In this large international cohort of children genetically at risk for type 1 diabetes followed for more than 8 years, we confirmed prior data showing that the risk of developing multiple autoantibodies was lower when children were born to mothers with type 1 diabetes compared with those having an affected father [23] or an affected sibling [24]. We were able to search for potential differences in demographics or patterns of autoimmunity that could help explain this difference. We observed no difference in the type of autoantibody appearing first between the three groups.

Despite differences in birth demographics among these three groups of relatives, this differential risk was not related to birthweight or linear growth (height and BMI) in the first 24 months of life. In single correlations, the children born to a mother with type 1 diabetes did have a higher birthweight and lower birth length. Based on the prediction growth models, the children with higher birthweight and lower birth length grew faster in the first 24 months. Maternal diabetes had a negative but minimal impact on growth during the first 24 months. Hence, based on the growth models, we would have expected these children to have increased risk of developing multiple autoantibodies. Yet, the Cox proportional hazard stepwise multivariate modelling for time to multiple autoimmunity showed that although those with increased birth length and growth velocity developed multiple autoantibodies sooner, children born to a mother with type 1 diabetes were less likely to develop multiple autoantibodies. Because of the differing results of the single correlations and the two predictive models, we conclude that the risk related to family history was not explained by differences in birthweight or growth during the first 2 years between the groups.

Three studies that specifically evaluated differences in number and type of measured autoantibodies in offspring of mothers compared with fathers with type 1 diabetes also reported that children with a father with type 1 diabetes were more likely to develop multiple autoantibodies than those with a mother with diabetes [23, 25, 26]. In contrast to our TRIGR study, these three studies did not account for HLA type risk stratification and therefore included both high and lower risk HLA groups. The similar finding of significantly higher rates of multiple autoantibodies when the father had type 1 diabetes in our cohort of young children with a first-degree relative with type 1 diabetes and increased HLA-conferred genetic risk suggests that HLA-conferred genetic risk also is not the explanation of the observed differences.

Although different rates of progression to type 1 diabetes have been reported depending on which autoantibody appears first [27], we observed no specific difference in the first-appearing antibody according to type for a first-degree relative with diabetes in this large cohort. The German BABY-DIAB study [26] also reported no specific order in which autoantibody developed first according to which relative had diabetes. Hence this lower risk associated with being born to a mother with type 1 diabetes is not explained by a differing pattern in which antibody appears first.

Multiple studies, including a recent meta-analysis, have reported a link between greater birthweight and the risk of type 1 diabetes [8] or islet cell autoimmunity [10]. In contrast, Bonifacio et al [25] reported that in infants of a mother with type 1 diabetes, those in the lower and upper tertiles for birthweight as well as the presence of a moderately elevated third-trimester maternal HbA_1c_, had a lower risk of autoimmunity to beta cells. In our cohort, children of mothers with type 1 diabetes had greater birthweight and an earlier gestational age but no relationship was shown between their birthweight and risk of multiple autoantibodies. Hence, in our study, the relative protective effect provided by having a mother with type 1 diabetes, compared with a father or sibling with type 1 diabetes, seems not to be mediated through birthweight.

The accelerator hypothesis [28] suggests that the increasing incidence rate of type 1 diabetes is related to increasing rates of childhood obesity and insulin resistance. In the Australian BABY-DIAB study [11], weight and BMI *z* score were found to be associated with higher rates of islet autoimmunity. Rapid linear growth during infancy has been identified as a risk factor for the development of autoimmunity and type 1 diabetes [11–13, 29]. A recent report from the TEDDY cohort found an increased risk of progression from autoimmunity to type 1 diabetes with lower height growth in infancy but increased weight gain and height gain in early childhood [30]. Similarly, we found that increased birth length and linear height velocity *z* scores in the first 2 years of life were independently associated with the development of multiple autoantibodies. Although the TRIGR Study Group [31] had previously reported higher BMI at 5 years in Canadian girls born to a mother with type 1 diabetes, we did not find any significant differences in BMI at 24 months between the three groups based on the family history in the current analysis.

In this analysis, we also found a significant difference in the risk of developing type 1 diabetes in children born to mothers with type 1 diabetes compared with children with fathers or siblings with type 1 diabetes. Previously, the TRIGR Study Group [32] reported that there was no significant effect of type of family history on the development of type 1 diabetes in the TRIGR cohort. However, that analysis compared children with a parent only (mother and fathers were combined) to those with sibling only or those with an affected sibling and parent.

This study has some strengths. Although the TEDDY study group [33] recruited more than 8600 children, only about 10% were from a first-degree-relative family, which remains fewer than TRIGR (*n* = 2074). Our cohort, including 277 children who developed multiple beta cell autoantibodies, is one of the largest to date to explore the influence of type of family history on the development of islet cell autoimmunity. The inclusion criteria were based on presence of a first-degree relative with type 1 diabetes and genetic (HLA) risk stratification at birth in an international multicentre, rather than country-specific, study. Data on five different autoantibodies serially measured through a follow-up period allowed for new comparisons of both type and timing of autoantibodies between the three family history groupings.

Some limitations should be considered when interpreting our results. The study presented here is based on a posteriori analysis, which cannot allow us to fully differentiate HLA effect from family history effect. The intentional wide geographical and ethnic background of the study population may have introduced significant environmental variables that could have influenced the results. We only analysed growth data from the first 24 months of life. It is possible that later growth velocity has more impact on progression to type 1 diabetes. However, this analysis was beyond the scope of this paper. Another point to consider when comparing our results with those of other studies is the definition used for multiple antibodies: defined as two or more autoantibodies present in each sample of IAA, GADA, IA-2A, ICA and ZnT8A. Some studies define this as at least two different time points with positive results and others have not included ICA in this definition due to concerns of overlapping measurements with GADA and IA-2A. The definition used in this paper was set at the start of the TRIGR study and has been used consistently in all its publications. Hence, the results presented may not be comparable with those of studies wherein a different definition of multiple antibodies had been used.

In the TRIGR cohort, children born to a mother with type 1 diabetes had a slightly lower but statistically significant risk of developing beta cell autoimmunity than those born with a father or a sibling with type 1 diabetes. However, difference in risk of developing autoantibodies based on family history type was not linked to differences in birthweight or growth (height velocity or BMI within the first 2 years of life). Ongoing efforts should likely focus on other genetic and epigenetic risks factors, as well as immunological mechanisms, to further advance our ability to identify those at risk of developing multiple antibodies and progressing to type 1 diabetes.

## Electronic supplementary material

ESM (PDF 392 kb)

## Data Availability

The dataset used for these analyses can be requested by e-mailing TRIGR@epi.usf.edu.

## Abbreviations

GADA: GAD autoantibodies
GLM: General linear regression model
IAA: Insulin autoantibodies
IA-2A: Tyrosine phosphatase-related insulinoma-associated 2 molecule autoantibodies
ICA: Islet cell autoantibodies
TEDDY: The Environmental Determinants of Diabetes in the Young
TRIGR: Trial to Reduce IDDM in the Genetically at Risk
ZnT8A: Zinc transporter 8 autoantibodies
