# Supplementary material for: Association between family history, early growth and the risk of beta cell autoimmunity in children at risk for type 1 diabetes
Source: Diabetologia. 2020 Oct 7;64(1):119–28. doi: 10.1007/s00125-020-05287-1 (PMC7716821; doi:10.1007/s00125-020-05287-1)
Supplement: Supplementary file 1 — (PDF 392 kb) [file 125_2020_5287_MOESM1_ESM.pdf]

Electronic supplementary material

ESM Table 1: *General Linear Regression Models Predicting Height Velocity (cm/yr and z-score) by Family History* of type 1 diabetes *(mother vs father vs sibling; n=1772)*

Parameters	Univariate Models			Multivariate Model [c]		
				β	SE (β)	*p* value
**Model 1 (cm/yr)**						
Family history from mother	0.30	0.14	0.038	-0.54	0.15	<0.001
Family history from father	0.10	0.14	0.464	-0.14	0.14	0.327
Gestational age (weeks)	-0.11	0.03	<0.001	-0.06	0.03	0.069
Northern European [a]	0.56	0.11	<0.001	0.31	0.10	0.002
Central Europe I [a]	0.26	0.14	0.064	0.47	0.13	<0.001
Male	0.23	0.09	0.012	0.39	0.08	<0.001
Vaginal delivery	0.10	0.09	0.260	0.18	.0.09	0.045
First diet other infant formula [b]	-5.55	1.93	0.004	-3.70	1.70	0.029
Birth weight (kg)	-0.56	0.08	<0.001	0.59	0.10	<0.001
Birth length (cm)	-0.32	0.02	<0.001	-0.38	0.02	<0.001
Duration of breast feeding	-0.04	0.02	0.117	-0.05	0.02	0.013
Treatment group assignment casein hydrosylate formula	0.03	0.09	0.773	-0.01	0.08	0.900
Birth order	-0.12	0.05	0.012	-0.13	0.05	0.004

**Model 2 (z-score)**						
Family history from mother	0.07	0.04	0.136	-0.16	0.04	<0.001
Family history from father	0.12	0.05	0.014	-0.04	0.04	0.362
Gestational age (weeks)	-0.05	0.01	<0.001	-0.02	0.01	0.070
Northern Europe [a]	0.17	0.04	<0.001	0.09	0.03	0.001
Central Europe II [a]	0.005	0.05	0.930	0.07	0.04	0.124
Central Europe I [a]	0.07	0.05	0.147	0.16	0.04	<0.001
Vaginal delivery	0.02	0.03	0.519	0.05	0.03	0.072
First diet other infant formula	-1.73	0.62	0.006	-1.03	0.49	0.035
Birth weight (kg)	-0.31	0.03	<0.001	0.17	0.03	<0.001
Birth length (cm)	-0.14	0.004	<0.001	-0.16	0.01	<0.001
Duration of breast feeding	-0.01	0.007	0.143	-0.02	0.01	0.010
Treatment group assignment casein hydrosylate formula	0.01	0.03	0.790	-0.01	0.02	0.726
Birth order	-0.05	0.015	0.001	-0.04	0.01	0.002

[a] Region were defined as Northern Europe (Finland, Sweden), Central Europe I (Czech Republic, Estonia, Hungary, Poland), Central Europe II (Germany, Luxembourg, The Netherlands, Switzerland), Southern Europe (Italy, Spain), USA, Canada or Australia

[b] First diet defined as breast milk, study formula, Nutramigen, other infant formula or other milk, or a combination of these (2 types of milk) received within the first 3 days of life

[c] Intercepts [β (SE (β), *P* value)] - Model 1 = 38.29 (1.37, <0.001), Model 2 = 8.15 (0.40, <0.001)

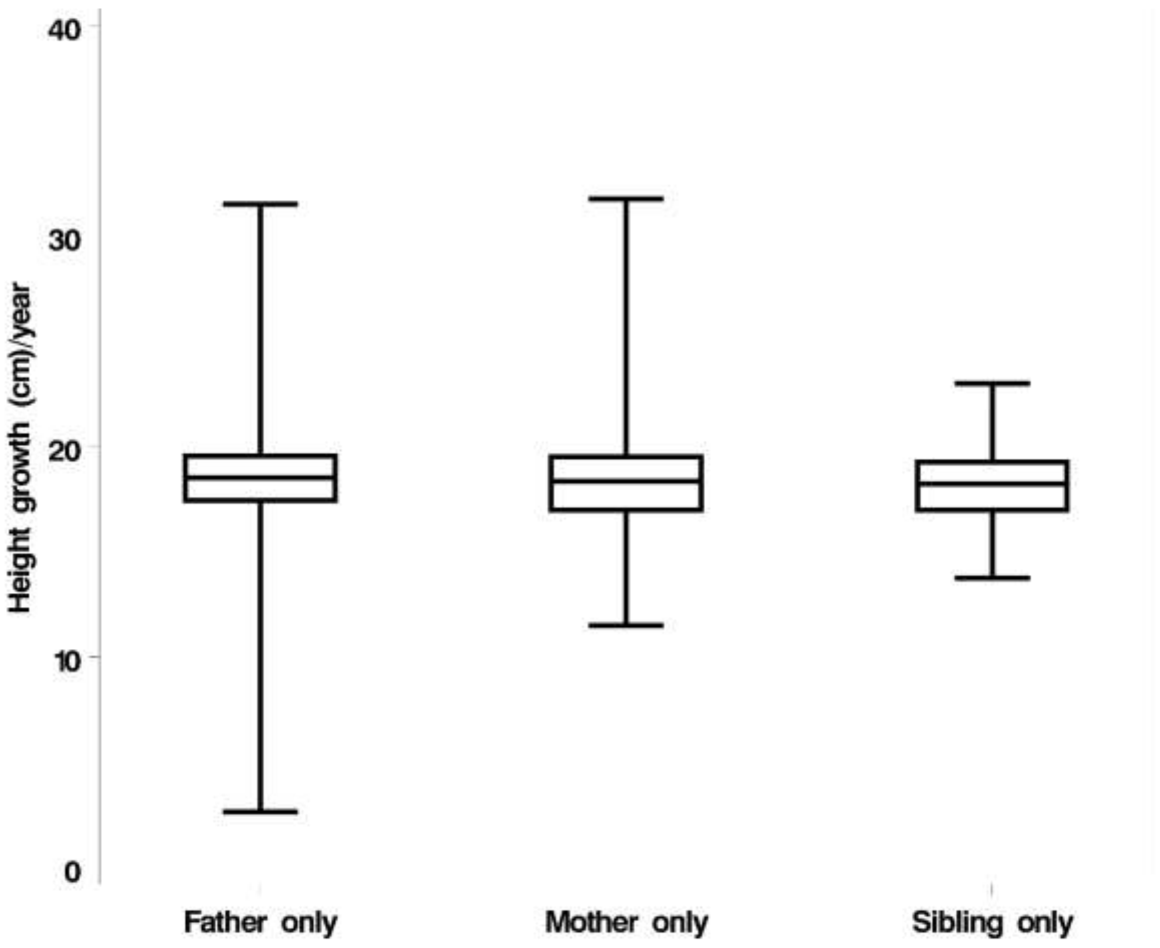

ESM Figure 1 – Difference in BMI (z-score) at 2 years of age by family history [mother (n=877) vs father (n=645) vs sibling (n=250)] (*p* = 0.054

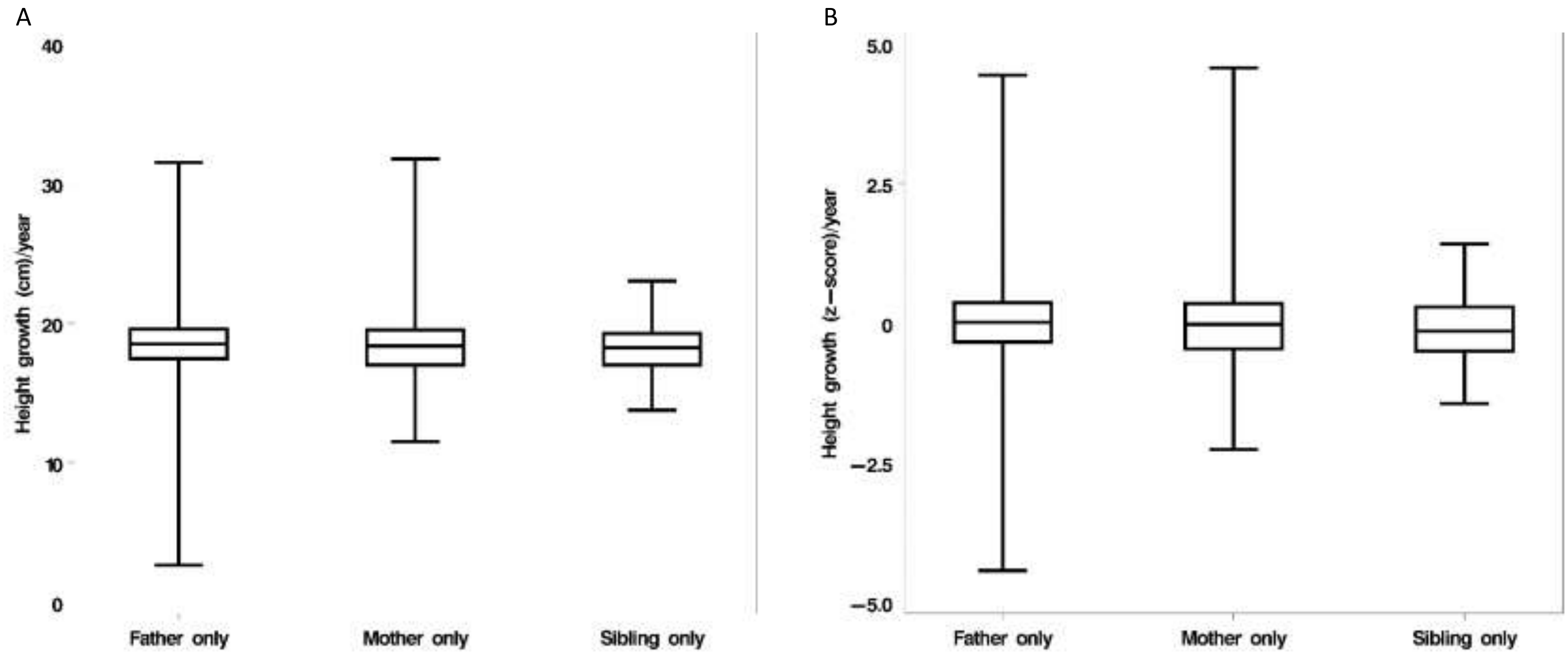

ESM Figure 2 – Difference in height velocity during the first 2 years of life by family history [mother (n=877) vs father (n=645) vs sibling (n=250)]. a) velocity in cm/yr, (*p* = 0.053) b) velocity as z-score/yr (*p* = 0.041

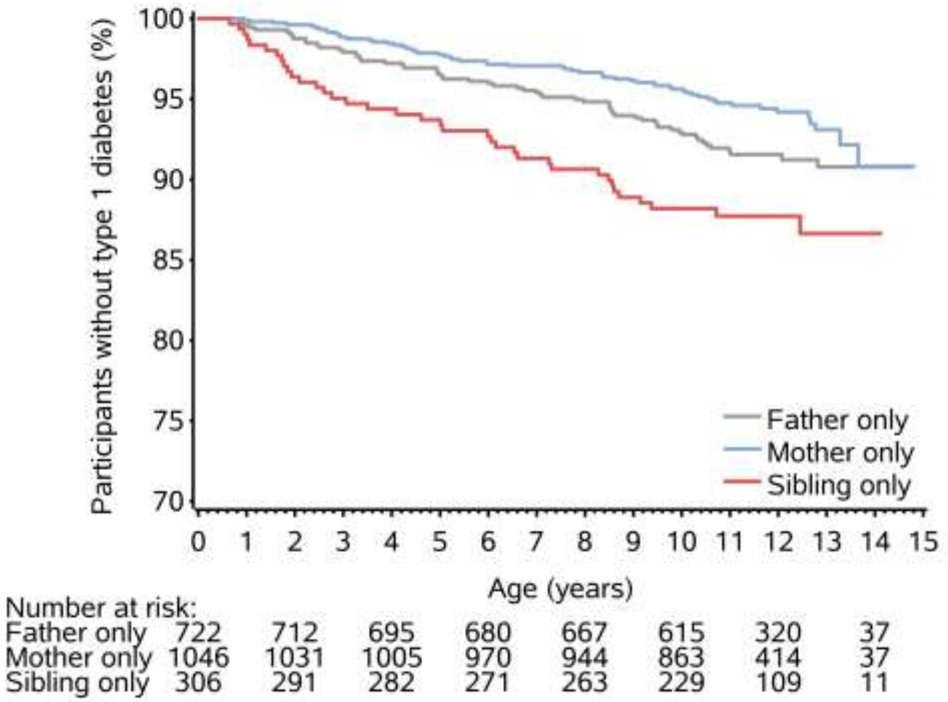

ESM Figure 3 – Time to type 1 diabetes by family history (mother vs father vs sibling), (Log-Rank Chi-Square 17.2; $p < 0.001$)

LIST OF TRIGR INVESTIGATORS FOR PUBLICATIONS / version September 2020

Administration/ Country	Study center	Last name	First name	Position
**Data Safety Monitoring Board**		Mandrup-Poulsen,	Thomas	Chair University of Copenhagen, Copenhagen, Denmark
		Arjas	Elias	Member, University of Helsinki, Helsinki, Finland
		Läärä	Esa	Member, University of Oulu, Oulu, Finland
		Lernmark	Åke	Member, University of Lund, Malmö, Sweden
		Schmidt	Barbara	Member, University of Pennsylvania, Philadelphia, PA, USA
		Krischer	Jeffrey P.	Observer, University of South Florida. Tampa FL, USA
**International Coordinating Center (ICC), University of Helsinki, Helsinki, Finland**		Åkerblom	Hans K. † *	PI of the Study until 30.6.08, Deputy PI from 1.7.2008
		Hyytinen	Mila	European Study Monitor
		Knip	Mikael	Deputy PI until 30.6.2008, PI of the Study from 1.7.2008, National Investigator
		Koski	Katriina	European Study Monitor
		Koski	Matti	IT Specialist
		Pajakkala	Eeva	European Study Monitor
		Salonen	Marja	Study Coordinator
**Data Management Unit (DMU), University of South Florida, Tampa, Florida, USA**		Cuthbertson	David	Biostatistician
		Krischer	Jeffrey P.	PI of the DMU
		Shanker	Linda	Coordinator

Administration/ Country	Study center	Last name	First name	Position
**Canadian Coordinating Center, University of Western Ontario, London, and University of Ottawa, Ontario**		Bradley	Brenda	National Coordinator
		Dosch	Hans-Michael	Co-Investigator for Canada
		Dupré	John	Co-PI for North America and National Investigator until 08/12/2015, Executive Committee
		Fraser	William	Co-Investigator for Canada Executive Committee
		Lawson	Margaret	Co-Investigator for Canada Executive Committee
		Mahon	Jeffrey L.	Co-PI for North America and National Investigator after 08/12/2015, Executive Committee
		Sermer	Mathew	Co-Investigator for Canada, Executive Committee
		Taback	Shayne P.	Co-Investigator for Canada, Executive Committee
**USA Coordinating Center, University of Pittsburgh, Pennsylvania and University of Washington, Seattle, Washington**		Becker	Dorothy	Co-PI for North America, National Investigator, Executive Committee
		Franciscus	Margaret	National Coordinator
		Nucci	Anita	National Coordinator, Nutrition Coordinator of North America
		Palmer	Jerry	Executive Committee

Administration/ Country	Study center	Last name	First name	Position
**Nutritional Epidemiology Unit, National Institute for Health and Welfare, Helsinki, Finland**		Virtanen	Suvi M.	Head of Nutritional Epidemiology Unit
**Australia**	**AUS01 – Westmead, New South Wales - Children's Hospital**	Catteau<br>Howard	Jacki<br>Neville	National Coordinator<br>National Investigator
	**AUS02 – Newcastle, New South Wales - John Hunter Children's Hospital**	Crock	Patricia	Local Investigator
	**AUS03 – Sydney, New South Wales - Sydney Children's Hospital**	Craig	Maria	Local Investigator
**Canada**	**CAN01 – London, Ontario - St. Joseph's Health Care Centre**	Clarson<br>Bere	Cheril L.<br>Lynda	Local Investigator<br>Coordinator
	**CAN02 – Vancouver, British Columbia - Children's and Women's Health Centre of British Columbia**	Thompson<br>Metzger<br>Marshall<br>Kwan	David<br>Daniel<br>Colleen<br>Jennifer	Local Investigator<br>Local Investigator<br>Coordinator (In Transition)<br>Coordinator (In Transition)
	**CAN03 – Calgary, Alberta - Alberta Children's Hospital**	Stephure<br>Pacaud<br>Schwarz	David K.<br>Daniele<br>Wendy	Local Investigator<br>Co-Investigator<br>Coordinator

Administration/ Country	Study center	Last name	First name	Position
**Canada**	**CAN04 – Edmonton, Alberta - Walter MacKenzie Health Sciences**	Girgis<br>Thompson	Rose<br>Marilyn	Local Investigator<br>Coordinator
	**CAN05 – Winnipeg, Manitoba - Health Sciences Centre**	Taback<br>Catte	Shayne P<br>Daniel	Local Investigator<br>Coordinator
	**CAN06 – Ottawa, Ontario - Children's Hospital of Eastern Ontario and The Ottawa Hospital**	Lawson<br>Bradley	Margaret L<br>Brenda	Local Investigator<br>Coordinator
	**CAN07 – Toronto, Ontario - Mount Sinai Hospital/Hospital for Sick Children**	Daneman<br>Sermer<br>Martin	Denis<br>Mathew<br>Mary-Jean	Local Investigator<br>Co-Investigator<br>Coordinator
	**CAN08 – Quebec, Quebec - CHUQ**	Morin<br>Frenette<br>Ferland	Valérie<br>Line<br>Suzanne	Local Investigator<br>Co-Investigator<br>Coordinator
	**CAN09 - Saint John, New Brunswick – Regional Hospital**	Sanderson<br>Heath	Susan<br>Kathy	Local Investigator<br>Coordinator
	**CAN10 – Montreal, Quebec - L' Hôpital Sainte-Justine**	Huot<br>Gonthier<br>Thibeault	Céline	Local Investigator<br>Co-Investigator<br>Coordinator
	**CAN11 – Montreal, Quebec - Children's Hospital**	Legault<br>Laforte	Laurent<br>Diane	Local Investigator<br>Coordinator

Administration/ Country	Study center	Last name	First name	Position
**Canada**	**CAN13 - St. John's, Newfoundland and Labrador - Janeway Child Health Center**	Bridger<br>Crummell	Tracey<br>Cheryl	Local Investigator<br>Coordinator
	**CAN14 – Kingston, Ontario - Kingston General Hospital/ Queen's University**	Houlden<br>Breen	Robyn<br>Adriana	Local Investigator<br>Coordinator
	**CAN15 – Regina, Saskatchewan - Regina Qu'Appelle**	Carson<br>Kelly	George<br>Sheila	Local Investigator<br>Coordinator
	**CAN16 – Saskatoon, Saskatchewan - Royal University Hospital**	Sankaran<br>Penner	Koravangattu<br>Marie	Local Investigator<br>Coordinator
	**CAN17 – Peterborough, Ontario - Peterborough Regional Health Centre**	White<br>King	Richard A<br>Nancy	Local Investigator<br>Coordinator
	**CAN18 – Victoria, British Columbia - Vancouver Island Health Research Centre**	Popkin<br>Robson	James<br>Laurie	Local Investigator<br>Coordinator
**Czech Republic**	**CZE01 - Prague - Faculty Hospital Kralovske Vinohrady**	Al Taji<br>Mendlova<br>Romanova<br>Vavrinec<br>Vosahlo	Eva<br>Pavla<br>Martina<br>Jan † *<br>Jan	National Investigator/Coordinator<br>National Coordinator<br>Co-Investigator<br>National Investigator<br>Co-Investigator

Administration/ Country	Study center	Last name	First name	Position
**Czech Republic**	**CZE02 - Brno - Hospital Milosrdnych Bratri**	Brazdova	Ludmila	Local Investigator
	**CZE03- Olomouc - Faculty Hospital Olomouc**	Venhacova<br>Venhacova	Jitrenka<br>Petra	Local Investigator<br>Co-Investigator
	**CZE04 - Usti nad Labem - Hospital of Masryk**	Cipra	Adam	Local Investigator
	**CZE05 - Ceske Budejovice - Hospital Ceske Budejovice**	Tomsikova	Zdenka	Local Investigator
	**CZE06 - Plzen - Faculty Hospital Plzen**	Paterová	Petra	Local Investigator
	**CZE07 - Zlin - Hospital of Bata**	Gogelova	Pavla	Local Investigator
**Estonia**	**EST01 - Tallinn - Tallinn Children's Hospital**	Einberg<br>Riikjärv	Ülle<br>Mall-Anne	Co-Investigator<br>Local Investigator
	**EST02 - Tartu - Tartu University Children's Hospital**	Ormisson<br>Tillmann	Anne<br>Vallo	National Investigator<br>Co-Investigator

Administration/ Country	Study center	Last name	First name	Position
**Finland**	**FIN01 - Helsinki – Children's Hospital, University of Helsinki**	Johansson	Susanne	National Coordinator
		Kleemola	Päivi	National Coordinator
		Parkkola	Anna	Local Investigator
	**FIN02 - Helsinki - Department of Obstetrics and Gynecology, University of Helsinki**	Järvenpää	Anna-Liisa	Local Investigator
	**FIN03 - Espoo - Jorvi Hospital**	Hämäläinen	Anu-Maaria	Local Investigator
		Kiiveri	Sanne	Local Investigator
	**FIN04 - Kotka - Kymenlaakso Central Hospital**	Salonen	Maria	Local Investigator
		Tenhola	Sirpa	Local Investigator
	**FIN05 - Lahti - Paijat-Hame Central Hospital**	Salonen	Pia	Local Investigator
	**FIN06 - Tampere - Department of Pediatrics, Tampere University Hospital**	Jason	Eeva	Local Investigator
		Selvenius	Jenni	Local Investigator
		Siljander	Heli	Co-Investigator
	**FIN07 - Pori - Satakunta Central Hospital**	Ylitalo	Samuli	Local Investigator
	**FIN08 - Jyväskylä - Central Finland Central Hospital**	Paajanen	Ilkka	Local Investigator
	**FIN09 - Seinäjoki - South Ostrobotnia Central Hospital**	Talvitie	Timo	Local Investigator

Administration/ Country	Study center	Last name	First name	Position
	**FIN10 - Hyvinkää - Hyvinkää Hospital**	Lindström	Kaija	Local Investigator
	**FIN11 - Kuopio - Department of Pediatrics, Kuopio University Hospital**	Huopio<br>Pesola	Hanna<br>Jouni	Local investigator<br>Co-Investigator
	**FIN12 - Oulu - Department of Pediatrics, Oulu University Hospital**	Veijola<br>Tapanainen	Riitta<br>Päivi	Local Investigator<br>Co-Investigator
	**FIN13 - Hämeenlinna - Kanta-Hame Central Hospital**	Alar	Abram	Local Investigator
	**FIN14 - Vaasa - Vaasa Central Hospital**	Popov	Erik	Local Investigator
	**FIN15 - Lappeenranta - South Carelian Central Hospital**	Virransalo	Ritva	Local Investigator
	**FIN16 - Mikkeli - Mikkeli Central Hospital**	Nykänen	Päivi	Local Investigator

Administration/ Country	Study center	Last name	First name	Position
**Germany**	**GER01 - Hannover - Kinder- und Jugendkrankenhaus – Auf der Bult**	Aschemeier	Bärbel	National Coordinator
		Danne	Thomas	National Investigator
		Kordonouri	Olga	Co-Investigator
**Hungary**	**HUN01 - Budapest - Semmelweis Medical University**	Krikovszky	Dóra	Co-Investigator
		Madácsy	László	National Investigator
**Italy**	**ITA01 - Rome - University Campus Bio-Medico of Rome**	Khazrai	Yeganeh Manon	Local Coordinator
		Maddaloni	Ernesto	Local Coordinator
		Pozzilli	Paolo	National Investigator
	**SAR01 - Cagliari - St. Michele Hospital**	Mannu	Carla	Local Coordinator
		Songini	Marco	National Investigator
**Luxembourg**	**LUX01 - Luxembourg - Centre Hospitalier de Luxembourg**	de Beaufort	Carine	National Investigator
		Schierloh	Ulrike	Co-Investigator
**The Netherlands**	**NET01 - Rotterdam - Sophia Children's Hospital**	Bruining	Jan † *	National Investigator
		Bisschoff	Margriet	National Coordinator
**Poland**	**POL01 - Wroclaw - Medical University of Wroclaw**	Basiak	Aleksander	Co-Investigator
		Wasikowa	Renata	National Investigator
	**POL02 - Krakow - Polish-American Children's Hospital**	Ciechanowska	Marta	Local Investigator

Administration/ Country	Study center	Last name	First name	Position
**Poland**	**POL03 - Katowice - Medical University of Silesia**	Deja	Grazyna	Co-Investigator
		Jarosz-Chobot	Przemyslawa	Local Investigator
	**POL04 - Lodz - Medical University of Lodz**	Szadkowska	Agnieszka	Co-Investigator
	**POL05 - Lodz - Polish Mother's Memorial Hospital (I.C.Z.M.P)**	Cypryk	Katarzyna	Local Investigator
		Zawodniak-Szalapska	Malgorzata	Co-Investigator
**Spain**	**SPA01 - Cruces University Hospital-UPV/EHU-CIBERDEM/CIBERER, Barakaldo, Spain**	Castano	Luis	National Investigator
		Chueca	Maria	Co-Investigator
		Gonzalez Frutos	Teba	Local Coordinator
	**SPA02 - Madrid - Hospital Clinico San Carlos**	Serrano-Ríos	Manuel	National Investigator
		Martínez-Larrad	María Teresa	Local Coordinator
		Hawkins	Federico Gustavo	Co-Investigator
	**SPA03 - Madrid - Hospital Gregorio Marañon**	Rodriguez Arnau	Dolores	Co-Investigator
**Sweden**	**SWE01 - Linköping - University of Linköping**	Ludvigsson	Johnny	National Investigator
		Smolinska Konefal	Malgorzata	National Coordinator
	**SWE02 - Uddevalla - Uddevalla Hospital**	Hanas	Ragnar	Local Investigator
	**SWE03 - Göteborg - GothenburgThe Queen Silvia Children's Hospital**	Lindblad	Bengt	Local Investigator

Administration/ Country	Study center	Last name	First name	Position
**Sweden**	**SWE05 - Halmstad - Halmstad Hospital**	Nilsson	Nils-Östen	Local Investigator
	**SWE06 - Trollhättan - Trollhättan Hospital**	Fors	Hans	Local Investigator
	**SWE07 - Norrköping - Vrinnevi Hospital**	Nordwall	Maria	Local Investigator
	**SWE08 - Borås - Borås Hospital**	Lindh	Agne	Local Investigator
	**SWE09 - Karskrona - Karlskrona Hospital**	Edenwall	Hans	Local Investigator
	**SWE10 - Örebro - University Hospital**	Åman	Jan	Local Investigator
	**SWE11 - Jönköping - Ryhovs Hospital**	Johansson	Calle	Local Investigator
**Switzerland**	**SWT01 - Zürich - University Children's Hospital**	Gadient	Margrit	Local Coordinator
		Konrad	Daniel	National Investigator
		Schoenle	Eugen	National Investigator
**USA**	**USA01 – Pittsburgh, Pennsylvania - Children's Hospital of Pittsburgh**	Becker	Dorothy	USA National Investigator / Pittsburgh Local Investigator
		Daftary	Ashi	Co-Investigator
		Klein	Mary Beth	Pittsburgh Coordinator
		Gilmour	Carol	Co-Investigator

Administration/ Country	Study center	Last name	First name	Position
**USA**	**USA02 – Seattle, Washington - VA Puget Sound Health Care System and University of Washington**	Palmer	Jerry	Local Investigator
		Palmer	Patty	Local Investigator
		Malone	Patty	Coordinator
	**USA03 - St. Louis, Missouri - Washington University**	Tanner-Blasiar	Marilyn	Coordinator
		White	Neil	Local Investigator
	**USA04 - Los Angeles, California - Mattel Children's Hospital of UCLA**	Devaskar	Uday	Local Investigator
		Horowitz	Heather	Coordinator/dietitian
		Rogers	Lisa	Coordinator/dietitian
	**USA05 – Ponce, Puerto Rico - Ponce School of Medicine**	Colon	Roxana	Coordinator
		Frazer	Teresa	Co-Investigator
		Torres	Jose	Local Investigator
	**USA06 - New York, New York - Naomie Berrie Diabetes Center**	Goland	Robin	Local Investigator
		Greenberg	Ellen	Coordinator
		Schachner	Holly	Co-Investigator
		Softness	Barney	Co-Investigator
**Laboratories**	**HLA-typing Laboratory – University of Turku, Turku – Finland**	Ilonen	Jorma	Head of HLA-typing Laboratory
	**HLA-typing Laboratory – University of Pittsburgh, Pennsylvania - USA**	Trucco	Massimo	Head of HLA-typing Laboratory
		Nichol	Lynn	Chief Technician

**Cow's Milk Antibody Laboratory – University of Helsinki, Helsinki – Finland**	Savilahti	Erkki	Head of Cow's Milk Antibody Laboratory
**Autoantibody Laboratory – University of Helsinki, Helsinki – Finland**	Härkönen	Taina	Co-Investigator
	Knip	Mikael	Head of Antibody Laboratory
**T-Cell Laboratory – Helsinki, Helsinki – Finland**	Vaarala	Outi	Head of T-cell Laboratory
	Luopajärvi	Kristiina	Co-Investigator
**T-Cell Laboratory – Hospital for Sick Children, Toronto, Ontario - Canada**	Dosch	Hans-Michael	Head of T-Cell Laboratory
